# Different stress-related phenotypes of BALB/c mice from in-house or vendor: alterations of the sympathetic and HPA axis responsiveness

**DOI:** 10.1186/1472-6793-10-2

**Published:** 2010-03-09

**Authors:** Jakob Olfe, Grazyna Domanska, Christine Schuett, Cornelia Kiank

**Affiliations:** 1 Department of Immunology, DFG graduate school GK840, Ernst-Moritz-Arndt-University Greifswald, Germany; 2 David Geffen School of Medicine, UCLA, VA Greater Los Angeles Healthcare System, Digestive Diseases Research Center and Center for Neurobiology of Stress, Digestive Diseases Division, Department of Medicine, Los Angeles, CA, USA

## Abstract

**Background:**

Laboratory routine procedures such as handling, injection, gavage or transportation are stressful events which may influence physiological parameters of laboratory animals and may interfere with the interpretation of the experimental results. Here, we investigated if female BALB/c mice derived from in-house breeding and BALB/c mice from a vendor which were shipped during their juvenile life differ in their HPA axis activity and stress responsiveness in adulthood.

**Results:**

We show that already transferring the home cage to another room is a stressful event which causes an increased HPA axis activation for at least 24 hours as well as a loss of circulating lymphocytes which normalizes during a few days after transportation. However and important for the interpretation of experimental data, commercially available strain-, age- and gender-matched animals that were shipped over-night showed elevated glucocorticoid levels for up to three weeks after shipment, indicating a heightened HPA axis activation and they gained less body weight during adolescence. Four weeks after shipment, these vendor-derived mice showed increased corticosterone levels at 45-min after intraperitoneal ACTH challenge but, unexpectedly, no acute stress-induced glucocorticoid release. Surprisingly, activation of monoaminergic pathways were identified to inhibit the central nervous HPA axis activation in the vendor-derived, shipped animals since depletion of monoamines by reserpine treatment could restore the stress-induced HPA axis response during acute stress.

**Conclusions:**

In-house bred and vendor-derived BALB/c mice show a different stress-induced HPA axis response in adulthood which seems to be associated with different central monoaminergic pathway activity. The stress of shipment itself and/or differences in raising conditions, therefore, can cause the development of different stress response phenotypes which needs to be taken into account when interpreting experimental data.

## Background

Laboratory animals such as mice and rats are bred under standardized conditions which are very different from their conspecifics in biological environments. It is known that reduced environmental stimuli in standardized breeding laboratories create a highly stress-responsive phenotype in laboratory animals which is often ignored [1,2]. Environmental stressors in laboratories include restraint, noise, temperature or the presence of the experimenter. Several studies have shown that routine laboratory procedures including handling, weighing, transportation, or orogastric gavages can significantly activate the neuroendocrine stress response [3-10].

A stimulation of the sympathetic nervous system leads to elevated levels of norepinephrine - mainly released from sympathetic nerves - and epinephrine - secreted from the adrenal medulla. The activation of the hypothalamus-pituitary-adrenal axis (HPA axis) during stress stimulates the release of glucocorticoids (GCs) from the adrenal cortex after adrenocorticotrope hormone (ACTH) is secreted from the anterior pituitary [11-13]. This is induced by hypothalamic stimulatory factors such as corticotropin releasing factor (CRF) or vasopressin which are released from axon terminals of CRF neurons originating in the parvocellular part of the paraventricular nucleus of the hypothalamus (pPVN). As a consequence of stress hormone release, glucose levels are increased, heart rate and blood pressure are elevated, and behavioural alterations become detectable [11-15]. Stress hormones are well known modulators of the immune system [14-18] and induce metabolic alterations such as increased glycogenolysis and gluconeogenesis as well as accelerated lipolysis, shift metabolic functions towards carbohydrate, lipid and protein catabolism [19-23]. Acute stress activates both the sympathetic nervous system and the HPA axis. First, within one to five minutes a strong increase in plasma catecholamine concentration is observed, whereas the maximal release of ACTH is found within five to 15 minutes which is followed by an elevation of the plasma GC concentration about 15 min later. After the termination of an acute stress exposure the neuroendocrine systems progressively return to pre-stress levels within 24-72 hours.

Besides influences of age and gender [24,25], it was shown that different rodent strains vary in their stress-reactive phenotype, with high variations in their HPA axis activation or immune response. We recently showed that BALB/c mice are highly stress sensitive compared with CBA mice [24] and show less stress-induced disturbances compares with C57BL/6 mice after repeated combined acoustic and restraint stress (unpublished).

Recently, it was shown that Sprague-Dawley rats which were obtained from different vendors have a different neuroendrocrine and immune response to lipopolysaccharide, interleukin-1β and turpentine [26,27]. This indicates that besides genetic differences, environmental factors determine the stress responsiveness in rodents.

During our own experiments we became aware of different stress response phenotypes of in-house bred female BALB/c mice and commercially available, gender- and age-matched animals that were shipped to our laboratory. Therefore, we here test the hypothesis that mice which were bred in the local Animal Vivarium differ in their stress-related HPA axis reactivity from mice that derived from a vendor which underwent over-night transportation in juvenile life.

## Results and Discussion

### Vendor-derived mice do not show a acute stress-induced glucocorticoid response

Changing the room or even shipment of laboratory animals are "normal" events prior to experiments with in-house bred or vendor-derived rodents. However, the influence of these procedures on the experimental outcome is less often taken into consideration.

**Figure 1:**
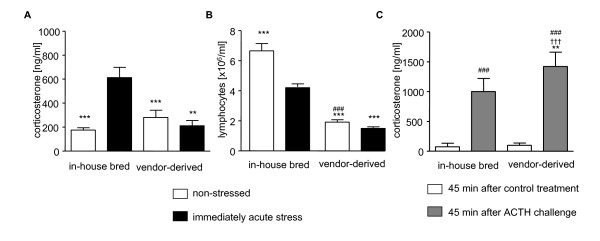
**Differences of stress responses of in-house-bred and vendor-derived, shipped mice**. **A, B**. Plasma corticosterone levels (A; ANOVA: F = 9.169; p < 0.001) and blood lymphocyte counts (B; ANOVA: F = 70.87; p < 0.001) of acutely stressed (black bars) and non-stressed mice (white bars) four weeks after room change (in-house bred) or shipment from vendor; n = 12 mice/group; summary of two independent experiments giving similar results. **C**. ACTH-induced (grey bars) and BSA control treatment-induced (white bars) plasma corticosterone levels measured four weeks after shipment from vendor or room change of in-house bred mice (ANOVA: F = 80.15; p < 0.001); n = 2 experiments; n = 6 mice/group; *p < 0.05, **p < 0.01, ***p < 0.001 compared with in-house bred, acutely stressed or ACTH-challenged group; ^###^p < 0.001 compared with in-house bred, non-stressed group; ^††^p < 0.01 compared with vendor-derived control group; comparison by Tukey test.

In our study, we transported the animals' home cages of in-house bred mice into a new room within our Animal Vivarium compared with vendor-derived mice that underwent a long-distance shipment. All animals were allowed to adapt to the same new environment for four weeks until acute stress experiments were performed. However, the experimental results of in-house bred vs. vendor-derived animals showed significant differences: Acute combined acoustic and restraint stress induced a corticosterone response in in-house bred animals whereas animals that were shipped four weeks prior the experiments did not show a stress-induced GC release (Fig. 1A). The animals of the shipped grouped showed by trend increased but not significantly higher baseline corticosteone levels compared with in-house bred mice (Fig. 1A). In general, the basal GC concentrations of all mice were quiet high compared with the observation of other authors which is typically lower than 100 ng/ml in a quiet environment [10,11,28,29]. In our studies, healthy female BALB/c mice in the "non-stressed" control group showed in average 176 ± 114.74 ng/ml (n = 36 mice, n = 4 experiments) which was reproducible throughout many experiments [23,24]. Whether this high baseline has any additional ceiling effect on stress reactivity in the group of the vendor-derived mice remains to be elucidated.

The animals derived from the vendor had significantly lower lymphocyte counts in the peripheral blood, even when non-stressed, compared with in-house bred control mice (Fig. 1B). However, a stress-induced lymphocytopenia was measurable in both in-house bred and vendor-derived animals (Fig. 1B).

It is known, that acute stress induces lymphocytopenia in rodents by changing the distribution pattern of T cell, NK cell, NKT cell and B cell populations from the circulation into peripheral organs such as the skin or lymph nodes and bone marrow [28-31]. In humans, a post-stress lymphocytopenia was shown after physical stress [32], during recovery period from exercise [33] and due to stress of surgery [34,35]. Both catecholamines and GCs were shown to mediate such leukocyte re-distribution after acute stress in rodents and humans [24,29,30,32,36,37].

Interestingly, mice which derived from the vendor did not show increased GCs levels after acute stress exposure but still displayed a loss of lymphocytes from the circulation. Therefore, we suggest that the lymphocytopenia in our stress experiments is mediated by activation of catecholaminergic pathways.

It is well established that GCs reveal a high potential to induce apoptosis of lymphocytes [for review see [38]] and, therefore, long-lastingly increased GCs levels in the vendor-derived animals may have induced lymphocyte apoptosis in these animals which resulted in lower lymphocyte numbers in the peripheral blood at 4 weeks after shipment.

We started to investigate whether the alterations of the stress-induced corticosteroid response in vendor-derived mice was mediated by adrenal insufficiency. Therefore, we performed an ACTH test. Mice that underwent the shipment four weeks prior to analysis had an exaggerated adrenal response to ip ACTH resulting in higher plasma corticosterone levels compared with animals of our own breeding facility (Fig. 1C). These data show that the lack of stress-induced GC-release in the vendor-derived mice is not related to adrenal insufficiency but rather may result from central HPA axis down-regulation. Interestingly, preliminary data of our group show that female BALB/c mice show a plateau-maximal corticosterone response between 30-60 min after ACTH application (see additional file 1). Whether the ACTH-induced exaggeration of corticosterone release in vendor-derived mice is also associated with a different kinetics remains to be elucidated.

The long-term influences of stress on adrenocortical GC secretion are controversial. Findings vary from showing no influence of stress on corticosterone release [39], to a peripheral desensitization of the ACTH and GC response at 1-4 weeks after a single immobilization stress [40] up to showing an increased ACTH responsiveness to novel environmental challenges [41,42]. In our current study, we show that up to four weeks after shipment the vendor-derived mice show an increased external ACTH-induced GC release indicating a peripheral sensitization of the adrenocortical response.

### Long-lasting alteration of HPA axis responsiveness after shipment of mice

There are several reports indicating that HPA axis deregulation after stress is time-dependent and varies depending on the stressor intensity [40,43,44].

We were interested whether changing the room or shipment differentially affect GC levels and how long these alterations will last. Therefore, we analyzed plasma GC levels of vendor-derived animals after 18 h over-night shipment in comparison with sex and age-matched animals of in-house breeding that only underwent a room change within their home cage prior to analysis. Samples were harvested 2 h after in-house transport or after over-night shipment, one day later, and weekly for the following three weeks. This time normally serves as an adaptation period under minimized stressful conditions [45-47] prior to the performance of repeated stress experiments as in our previously published studies [23,24,46-48].

**Figure 2:**
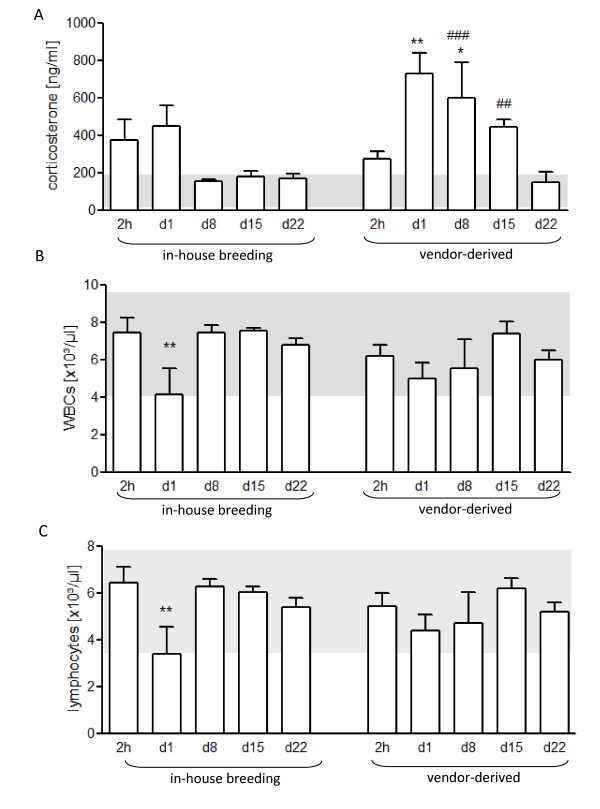
**Kinetics of glucocorticoid levels and lymphocyte counts after transportation stress**. **A**. Plasma corticosterone levels of female mice that were shipped for 18 h from vendor at the age of six weeks compared with gender and age-matched animals of in-house breeding that only underwent room change; measured immediately after transport, one day later and in the following three weeks (ANOVA for effects of breeding conditions F = 9.4, p < 0.0046; ANOVA for effects of time F = 6.03, p = 0.0011). **B, C**. Kinetics of total white blood cells (WBCs) and lymphocyte counts in the blood of mice after over-night transport from vendor compared with lymphocyte numbers of animals from own breeding facility (ANOVA for effects of breeding conditions F = 0.53, p = 0.4711; ANOVA for effects of time F = 3.02, p = 0.033); n = 4-20 mice/group *p < 0.05, **p < 0.01 comparison with basal levels of healthy control mice; ^##^p < 0.01, ^###^p < 0.001 comparision with vendor-derived mice at day 22; comparison by Turkey testing.

A room change of in-house bred animals induced a transient but not significant increase of GC concentrations in the plasma which lasted until the next day (Fig. 2A). This transient elevation of corticosteroid levels was associated with reduced numbers of blood lymphocytes (Fig. 2B). Surprisingly, mice that underwent the 18 h lasting shipment showed no heightened GC concentrations in the plasma 2 h after arrival. These animals, however, displayed increased plasma corticosterone levels one day later which remained elevated for the next two weeks (Fig. 2A). These vendor-derived mice only showed a transient and slight reduction of white blood cells (WBCs) counts in the blood one day after arrival (Fig. 2B) which was due to a significant loss of lymphocytes from the periphery (Fig. 2C). At day 22, the GC levels of these mice were finally comparable with those of in-house bred BALB/c mice (Fig. 2A).

When interpreting these data we need to consider that mice were anesthetized prior to blood collection by retroorbital puncture which is associated with handling and pain of injection and already can provoke a stress response of laboratory animals [3,4,7,10]. However, data of our own group (data not shown) and of others show that the neuroendocrine stress response to routine laboratory stressors such as cleaning a cage or transport needs 15 min to be established [49]. Narcosis and bleeding needed less than 10 min in our experiments and therefore, we believe that this short period of narcosis has a minor influence on the measured plasma corticosterone levels.

Short distance transportation and room change may be evaluated as mild stress whereas the over-night shipment of six-week-old mice is a more severe stressor. In fact, it was shown that acute severe stressors long-lastingly modulate the HPA axis as demonstrated in rats which showed a significantly increased ACTH response to a novel environment even two weeks after an initial single exposure to moderate electric footshock or immobilization stress [44,50]. Such a sensitization of the HPA axis was also inducible when animals were treated with a single dose of interleukin (IL)-1β [50,51]. Both footshock-induced and IL-1β-mediated increased HPA axis activation was mediated by a release of the neuropeptide arginine-vasopressin (AVP) from the external layer of the median eminence which potentiates the CRF induced release of ACTH from the pituitary gland [50,52]. Jansen et al. showed that a single injection of IL-1β (or amphetamine) decreased the innervation density of dopamine-β-hydroxylase (DBH) positive neurons as a marker of noradrenergic and adrenergic fibers in the PVN even three weeks after the injection especially in CRF neurons in rats [53]. In addition, Schmidt et al. recently showed that such a single administration of IL-1β to adult rats increases CRF and the CRF_1 _receptor mRNA expression in the PVN with a delay of one week after treatment which potentiates the secretion of ACTH and causes a long-term HPA sensitization [51]. In line with these data, Johnson et al. showed that a single session of tail shocks causes a sensitization of the HPA response to novel environments but also to immune stressors such as endotoxin (10 ng/g body weight) [43]. This was observed already at the first day and lasted for at least ten days - disappearing within 21 days after tailshock stress [43]. Importantly, this time kinetics closely resembles the plasma GC levels of vendor-derived animals in our experiments which normalized three weeks after arrival.

However, the data discussed until now do not explain why vendor-derived animals did not respond to acute psychological stress four weeks after transportation although their ACTH sensitivity of the adrenal gland was increased (Fig. 1C). Recently, it was shown that already a single restraint stress session can increase corticosterone levels for few weeks followed by a period of significantly reduced HPA responsiveness four weeks after the acute stress exposure [44,49]. This is in line with our observation that GC levels were initially increased after shipment and normalized later and also confirms our data of a reduced acute stress-induced HPA axis activation four weeks after shipment. Such a diminished responsiveness of the HPA axis to stress was assigned to a reduced hypothalamic CRF mRNA expression [44].

Thus, we hypothesize that shipment also exaggerates the HPA axis responsiveness for approximately three weeks due to central sensitization of CRF-releasing neurons in the pPVN followed by a phase of reduced CRF release four weeks after shipment that prevents an acute stress-induced release of GCs.

### Reduced gain of body weight in adolescence after shipment of mice

**Figure 3:**
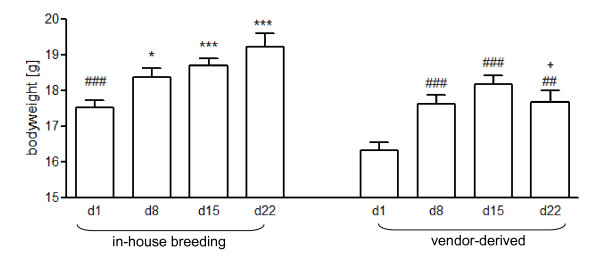
**Different body weight gain during adolescence of in-house bred and vendor-derived mice**. Kinetics of body weight gain 6-10 weeks after birth of mice that underwent 18 h-lasting over-night shipment from vendor compared with animals from own breeding facility, n = 4-20 mice/group. (ANOVA for effects of breeding conditions F(8) = 26.74, p < 0.0001; ANOVA for effects of time F(8) = 20.97, p < 0.0001); *p < 0.05, **p < 0.01, ***p < 0.001 compared to day 1 in the in-house bred group; ^#^p < 0.05, ^##^p < 0.01, ^###^p < 0.001 compared to day 1 in the vendor-derived group; ^+^p < 0.05 compared with day 22 in the in-house-bred group; comparison by Tukey testing.

Recently, we demonstrated that severe psychological stress causes a hypermetabolic syndrome which coincided with severe loss of body mass [23]. Therefore, we monitored the gain of body weight during adolescence in vendor-derived vs. in-house bred BALB/c mice. All animals showed an increase of body mass during the observation period (Fig. 3). However, already after arrival vendor-derived mice had a significantly lower body mass and did not catch up to the non-shipped mice until the age of 10 weeks (Fig. 3).

Earlier work shows that other stressors such as noise decrease the gain of body weight and food intake in young rats which may be mediated by HPA axis activation and norepinephrine release [54,55]. Thus, altered sensitivity of the stress axes as found in the vendor-derived vs. in-house bred mice very likely contribute to alterations in body weight gain.

### Lack of acute stress-induced GC response in vendor-derived mice is restored by reserpine treatment

Since many monoaminergic pathways including catecholaminergic and serotonergic signaling are known to modify the HPA axis activation in the brain, we used resperine which systemically depletes monoamines, to analyse their influences on the stress response in in-house bred vs. vendor-derived, shipped mice.

Reserpine long-lastingly inhibits the reuptake of monoamines from the synaptic cleft and depletes the transmitters from synaptic vesicles [56]. First, we showed that reserpine treatment was sufficient to deplete monoamines 24 h and 48 h after a single application of 1 μg resperine/g BW (see additional file 2).

**Figure 4:**
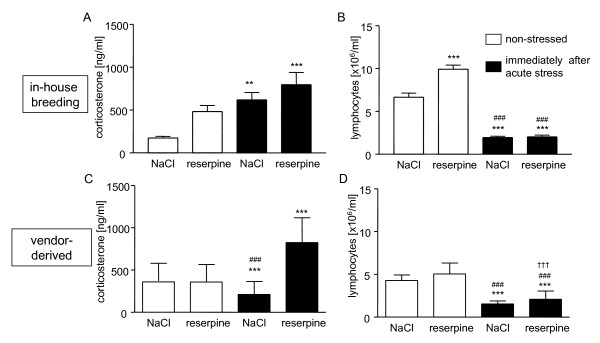
**Different acute stress response with or without reserpine treatment of in-house bred and vendor-derived mice**. Plasma corticosterone levels (A, ANOVA: F = 8.275, p = 0.0002) and lymphocyte counts of in-house bred (B, ANOVA: F = 114.6, p < 0.0001) as well as corticosterone levels (C, ANOVA: F(4) = 13.39, p < 0.0001) and lymphocyte counts in the blood of vendor-derived, shipped mice (D, ANOVA: F(4) = 42.48, p < 0.0001) immediately after acute stress exposure (black bars) compared with non-stressed animals (white bars). Mice were either pre-treated with reserpine in order to block sympathetic output during stress or control-treated with NaCl. n = 12 mice/group representative for two experiments giving similar results; *p < 0.05, **p < 0.01, ***p < 0.001 compared with NaCl treated, non-stressed mice, ^###^p < 0.001 compared with reserpine treated, non-stressed mice, ^†††^compared with NaCl treated, acutely stressed mice; comparison by Tukeys' test.

It is well known that catecholamines can activate the HPA axis by increasing CRF and ACTH release [57-59]. In our experiments, however, reserpine-induced depletion of catecholamines did not affect stress-induced GC release of in-house bred BALB/c mice (Fig. 4A) and did not impact stress-induced lymphocytopenia (Fig. 4B). The vendor-derived, shipped mice which were unable to establish an HPA axis response to acute psychological stress exposure (Fig. 1A, 4B), surprisingly, showed a restored stress-induced HPA axis activation (Fig. 4C) and also reduced lymphocytopenia after application of reserpine (Fig. 4D). This suggests that monoamines inhibit the activation of the HPA axis in these animals and indicates that acute stress-induced lymphocytopenia is, at least partially, mediated by catecholamine release.

Thus, our data indicates that monoamines can be involved in the central desensitization of the HPA axis and therefore, prevent an increase of corticosteroids after acute stress.

In contrast, other authors demonstrated that enhanced noradrenaline release causes stress sensitization which peaked after 1-3 weeks [51]. It is generally accepted that catecholamines which are derived from the nucleus tractus solitaris (NTS)-PVN pathway increase the expression of CRF mRNA, activate CRF releasing neurons, and facilitate the secretion of CRF into the portal blood [for review see [60]]. Schmidt et al. demonstrated a high morphological plasticity of the noradrenergic innervation of the pPVN during sensitization of the HPA axis for example after stress or single administration of IL-1β or amphetamines. They found decreased density of DBH positive neurons associated with an increased electrically-evoked release of noradrenaline from the pPVN [51].

Finally, there are few data on inhibitory effects of catecholaminergic influences on HPA axis activity showing a reduced HPA response to stress exposure when the noradrenergic reactivity of the bed nucleus of the stria terminalis (BNST) is enhanced or *vice versa *an increased HPA activity when the noradrenergic reactivity of the BNST is reduced [58,61]. The underlying mechanisms may be linked to direct GABAergic and non-GABAergic projections to the PVN and indirect projections through other hypothalamic nuclei [58,61].

The hypothesis that the catecholaminergic systems can also act inhibitory on the HPA axis is supported by findings of Radley et al. who showed that lesions of noradrenergic projections from the locus coeruleus to the medial prefrontal cortex in Sprague Dawley rats enhance emotional stress-induced ACTH release, which then stimulates GC release after restraint stress exposure. These authors propose that via this central neuronal pathway norepinephrine is involved in the adaptation response to stress by down-regulating HPA axis activity [62]. In addition, it was demonstrated that norepinephrine is able to inhibit central CRF release at higher concentrations [59]. Further studies are needed to analyze the short-term and long-term effects of monoamines on the regulation of the PVN and the modification of CRF and ACTH release under stressful conditions.

We hypothesize that stress adaptive inhibitory noradrenergic pathways interfere with acute psychological stress-induced HPA axis response in vendor-derived mice during the 4 weeks after shipment compared with in-house bred animals because monoamine depletion by reserpine disrupts this blockade and allows "normal" stress-induced GC release.

### Possible influence of environmental conditions on experimental outcomes

In our current study, we showed that shipment in the juvenile phase of life may significantly affect the stress responsiveness in adulthood. However, we do not know to what extent genotypic variations and differences of raising the animals in completely different environments in the postnatal period contribute to the development of different stress response phenotypes of in-house bred vs. vendor-derived mice. In 1999, Crabbe et al. showed that it makes no difference whether animals are in-house bred or shipped as adults five weeks before testing. The authors show that laboratory-specific differences and experimental protocols alter experimental data but, in contrast, genetic differences will not affect phenotypes significantly [63]. Finally, that raising conditions in the postnatal period influence the outcome of experiments is supported by recent publications which show that Sprague-Dawley from different vendors have a different neuroendrocrine and immune response to immune stimuli such as lipopolysaccharide, interleukin-1β and turpentine [26,27]. This indicates that besides genetic differences, environmental factors determine the stress responsiveness in rodents. Thus, environmental conditions specific to individual laboratories importantly influence experimental data and need to be taken into account. Since our experiments were performed in the same laboratory under equal environmental conditions, we hypothesize that shipment itself and/or difference in the breeding conditions in the pre- and postnatal period rather than laboratory conditions during the experiments or genetic differences in the analyzed animals contribute to the observed differences in modulating HPA axis and stress reactivity.

## Conclusions

Changing the room of laboratory animals transiently activates the stress axes without severely altering HPA activity and acute stress reactivity for the long-term. In turn, vendor-derived mice which were transported for 18 h show disturbed neuroendocrine regulatory circuits for at least 4 weeks and even impaired normal growth. This is associated with increased "basal" GC levels for about three weeks.

Moreover, the vendor-derived animals did not show corticosterone release during acute psychological stress which was linked to an altered central nervous HPA axis responsiveness because the adrenal gland was able to respond to exogenous ACTH. Reduced stress-induced GC release was connected to increased monoaminergic activity in the vendor-derived mice which is probably mediated by adaptive inhibitory central noradrenergic pathways.

Thus, routine laboratory procedures such as transportation potentially complicate the interpretation of data even when animals were allowed to adapt to the new environment. In addition, in-house bred and vendor derived animals may substantially differ in their stress responsive phenotypes. Therefore, experiments with laboratory animals should be planned carefully in order to prevent stress and the side effects of altered HPA axis activity, which in the worst case create non-comparable results and data.

## Methods

### Animals and housing conditions

In different experiments, inbred female BALB/cJ mice from our own breeding facilities or from Charles River Laboratories^® ^(Sulzfeld, Germany) were used at the age of 10-14 weeks. To reduced influences of genetic drift in the in-house bred mice, parental pairs for in house-breeding are constantly renewed with BALB/cJ mice derived from Charles River Laboratories^® ^(Sulzfeld, Germany). After weaning at the age of four weeks female and male animals were grouped six mice/cage and remained in these groups until the end of all experiments. Before starting the experiments both in-house bred and transhipped animals were transferred into a separate experimental room where they stayed in the same mouse incubator under acclimatized conditions (20-23°C, 50-55% humidity, 12/12 light cycle, lights on 6:00 AM). Food and water were available *ad libitum*. Mice were allowed to habituate to this environment for four weeks until experiments were performed. Influences of irregularities of the estrous cycles of unisexually grouped female mice [64] were not analyzed selectively and may cause higher standard deviations in the statistical analyses.

Animal experiments were approved by the State Animal Protection Committee of Mecklenburg-Vorpommern, Germany.

### Experimental procedures

#### Psychological stress model

Adult female BALB/c mice at the age of 10 weeks were exposed to a single session of combined acoustic and restraint stress (8-10 AM). Restraint stress was performed by placing mice in ventilated 50-ml conical tubes without penning their tail. Acoustic stress was induced by a randomized ultrasound emission device 19-25 kHz with 0-35 dB waves (SiXiS; Pat. NO. 109977, Taiwan). Non-stressed control mice were kept in their familiar environment, isolated from stressed animals and noise. Tissue and blood collection was performed alternating in all stressed and non-stressed animals immediately after ending the stress experiments in the morning (10-12 am) at the same setting (see below).

#### Analysis of effects of transport and shipment of laboratory animals

Six-week-old female BALB/cJ mice were obtained from Charles River^® ^laboratories (Sulzfeld, Germany) via overnight shipment (18 h transportation, 4:00 PM-10:00 AM). During this transportation 10-12 mice were animals grouped together and routinely bedded in ventilated boxes with free access to food and liquids. After arrival animals were grouped and placed in standard mouse cages (six mice/cage).

To characterize the effects of the shipment we used age-matched female BALB/c mice from in-house breeding for comparison, which only underwent a room change prior to analysis (in-house transportation). These animals were kept together in their home cages immediately after weaning at the age of four weeks (six mice/cage) until the end of the experiments. All in-house bred and vendor-derived animals were kept under minimal stress conditions in the same mouse incubator in our Animal Vivarium. Studies in stressed mice were controlled for reproducibility by repeating the experiments using animals from different transports/shipments.

Two hours after the arrival of the shipped animals or after in-house transport, analysis of plasma corticosteroid levels and lymphocyte counts were performed. This was also done one day later, as well as at days 8, 15, and 22 after in-house transport or shipment always in the morning between 8-10 AM. At the age of 10 weeks (4 weeks after in-house transport or shipment) animals underwent an ACTH test. To test the stress susceptibility of vendor-derived and in-house bred mice, we performed acute stress experiments with animals undergoing a single session of combined acoustic and restraint stress (8-10 AM).

#### Depletion of monoamines

Reserpine (Sigma, Steinheim, Germany) was used to deplete catecholamines, dopamine and serotonin. 20 μg of reserpine, which was diluted in acetic acid and 100 μl sterile physiological saline (from B. Braun Melsungen AG, Melsungen, Germany), was subcutaneously injected 24 h prior to an acute stress session. Control mice received 100 μl saline. Testing of the effectiveness of reserpine in blocking catecholamine release *in vivo *was performed in male BALB/c mice (Suppl. Mat. 2).

#### ACTH-test

50 ng/g bodyweight of adrenocorticotropin-fragment-1-24 (rat ACTH_1-24_, Sigma, Steinheim, Germany) diluted in 0.5% BSA/PBS solution or the BSA solution alone was injected subcutaneously. Previous studies of our own lab revealed that ACTH at this dose stimulates corticosterone release in healthy female BALB/c mice with a maximum plateau response at 30-60 min after challenge and then start to drop down (Suppl. Mat. 1). That's why we harvested blood at 45 min after ACTH or vehicle application in the current study. Animals were anesthetized and blood was harvested by retroorbital puncture. These studies were controlled for reproducibility by repeating the experiments using animals from different transports.

#### Blood collection

Blood was collected by retroorbital puncture in EDTA-blood collection tubes after anesthetizing animals by intraperitoneal injection with 120 μg/g BW Ketamin (Ratiopharm GmbH, Ulm, Germany) and 19 μg/g BW Rompun^® ^(Bayer AG, Leverkusen, Germany) diluted in pyrogen-free 0.9% physiological saline (Braun, Melsungen, Germany). Samples of all groups of one experiment were collected at the same day between 10-12 am. Plasma samples were immediately analyzed or stored at -20°C until use.

#### Measurement of corticosterone in the plasma

Plasma corticosterone levels were quantified by ELISA according to the instructions of the supplier (OCTEIA Corticosterone EIA, IDS, Boldon, UK).

#### Measurement of norepinephrine in the plasma

Plasma norepinephrine levels were quantified by ELISA according to the instructions of the supplier (IBL, Hamburg, Germany).

#### Quantification of white blood cells

Total numbers of white blood cells (WBCs) and subpopulations such as the lymphocyte population were measured with a hemocounter which allows the analysis of whole blood samples (Sysmex KX21N, Sysmex GmbH, Norderstedt, Germany), we used standard calibrations for human whole blood analysis.

### Statistical analysis

Statistical analyses was carried out with GraphPad Prism 5 (GraphPad Software Inc., San Diego, USA). Differences between two independent samples were analyzed by Mann-Whitney test. Data of more than two groups were statistically analyzed by Tukey testing after one-way ANOVA or two-way ANOVA in experiments with an additional time-dependent component. The data are depicted as bars showing means ± SEM; p < 0.05 was considered statistically significant.

## Abbreviations

ACTH: adrenocorticotropic hormone; BNST: bed nucleus of the stria terminalis; CRF: corticotropin releasing factor; DBH: dopamine-β-hydroxylase; GABA: gamma amino butteric acid; GCs: glucocorticoids; HPA axis: hypothalamus-pituitary-adrenal axis; mRNA: messenger ribonuclein acid; NTS: nucleus tractus solitaris; PVN: paraventricular nucleus of the hypothalamus; pPVN: parvocellular part of the paraventricular nucleus of the hypothalamus.

## Authors' contributions

JO carried out stress experiments and analyzed the stress-induced changes of body weight, hormone levels and immune state, performed immunoassays and performed statistical analysis. GD participated in planning, coordinate and performing the animal experiments. CS participated in the design and supervised of the study, offered funding to perform the study and helped to discuss and interpret the data. CK participated in the design and coordination of the study, participated in performing the animal experiments and performed statistical analysis, discussed and interpreted the data. All authors read and approved the final manuscript.

## Supplementary Material

Additional file 1 **ACTH-induced corticosterone response in BALB/c mice**. Intraperitoneal injection of ACTH induces a corticosterone response in female BALB/c mice. Plasma glucocorticoid concentrations are increased already 15 min and reach a plateau response between 30 min and 1 h after ip application of ACTH at 50 ng/g BW. Two and six hours after ACTH injection corticosterone levels were reduced compared wiht 30, 45 and 60 min measurements but still significantly elevated compared with basal levels. Twelve and 24 h after treatment glucocorticoid levels were back to normal; n = 4 mice/time point, reproducible in two independent experiments; *p < 0.05, **p < 0.01, ***p < 0.001 compared with basal levels. Click here for file

Additional file 2 **Depletion of monoamines by reserpine treatment**. Plasma norepinephrine (NE) levels of healthy male BALB/c mice which were pre-treated with reserpine in order to block sympathetic output were significantly reduced compared with NaCl treated control mice when measured 24 h and 48 h after i.p. injection (ANOVA: F = 11.93; p = 0.008); **p < 0.01 by Tukey testing. Click here for file
