# Supplementary figures and images for: Different stress-related phenotypes of BALB/c mice from in-house or vendor: alterations of the sympathetic and HPA axis responsiveness
Source: BMC Physiol. 2010 Mar 9;10:2. doi: 10.1186/1472-6793-10-2 (PMC2845127; doi:10.1186/1472-6793-10-2)

## Slide 1
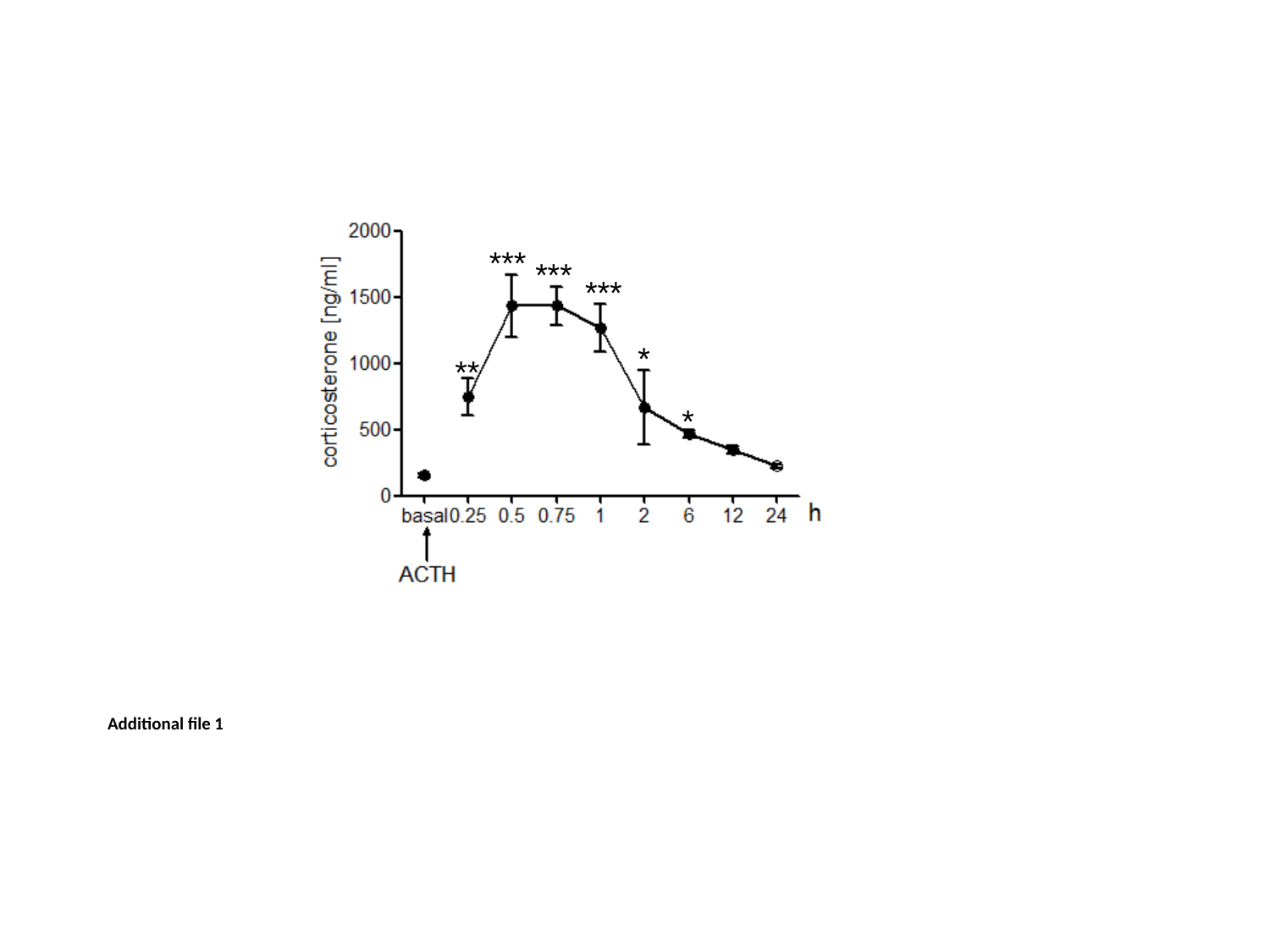

***
***
***
*
**
*
Additional file 1

Supplement: Additional file 1 — ACTH-induced corticosterone response in BALB/c mice. Intraperitoneal injection of ACTH induces a corticosterone response in female BALB/c mice. Plasma glucocorticoid concentrations are increased already 15 min and reach a plateau response between 30 min and 1 h after ip application of ACTH at 50 ng/g BW. Two and six hours after ACTH injection corticosterone levels were reduced compared wiht 30, 45 and 60 min measurements but still significantly elevated compared with basal levels. Twelve and 24 h after treatment glucocorticoid levels were back to normal; n = 4 mice/time point, reproducible in two independent experiments; *p < 0.05, **p < 0.01, ***p < 0.001 compared with basal levels. [file 1472-6793-10-2-S1.PPT]

## Slide 1
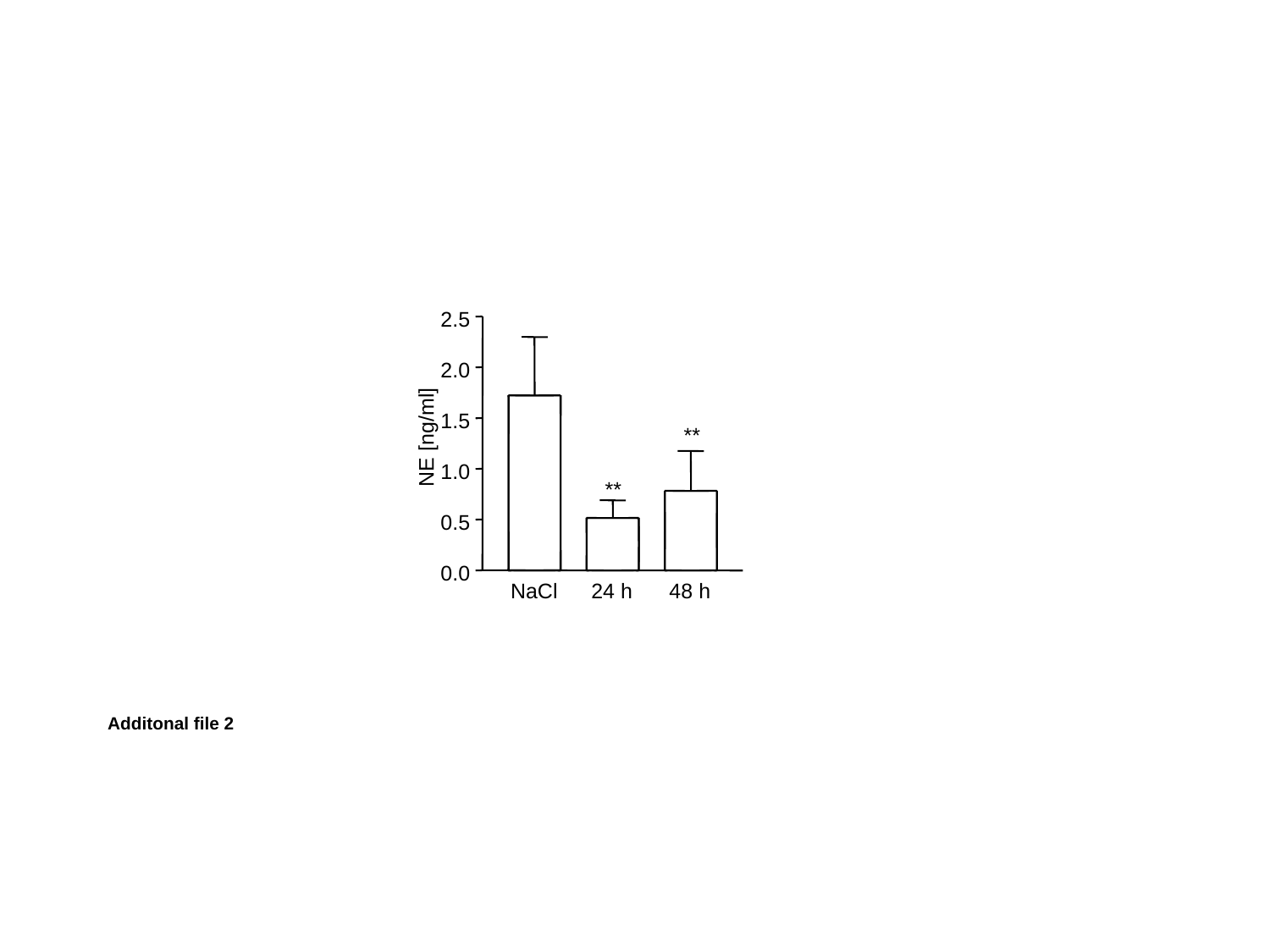

2.5
2.0
1.5
**
NE [ng/ml]
1.0
**
0.5
0.0
NaCl
24 h
48 h
Additonal file 2

Supplement: Additional file 2 — Depletion of monoamines by reserpine treatment. Plasma norepinephrine (NE) levels of healthy male BALB/c mice which were pre-treated with reserpine in order to block sympathetic output were significantly reduced compared with NaCl treated control mice when measured 24 h and 48 h after i.p. injection (ANOVA: F = 11.93; p = 0.008); **p < 0.01 by Tukey testing. [file 1472-6793-10-2-S2.PPT]
